# Exploring a new way to think about climate regions

**DOI:** 10.7554/eLife.67422

**Published:** 2021-03-16

**Authors:** Barnabas H Daru

**Affiliations:** Department of Life Sciences, Texas A&M University-Corpus ChristiCorpus ChristiUnited States

**Keywords:** climate classification, geographic signal, climate zones, vertebrates, Other

## Abstract

A new system for classifying climates emerges from modeling the environmental conditions that 26,000 species of tetrapods experience in their home range.

**Related research article** Calatayud J, Neuman M, Rojas A, Eriksson A, Rosvall M. 2021. Regularities in species' niches reveal the world's climate regions. *eLife* **10**:e58397. doi: 10.7554/eLife.58397

Most species are adapted to certain climates. Some are suited to the arid conditions of the Australian desert, whereas others prefer milder climates such as the temperate forests of North America. Variations in the Earth’s climate can be used to predict where in the world a species is most likely to inhabit (Woodward et al., 2004; Higgins et al., 2016). Identifying the impact climate has on biodiversity is important for understanding how different species will respond to global climate change (Bellard et al., 2012; Rock and Daru, 2021).

The Köppen system classifies the Earth’s climate into distinct zones based on the vegetation that grows in each region (Köppen, 1923). This system is based on the assumption that plant distributions are closely linked to climate (Olson et al., 2001). However, it is not clear if climate impacts the distribution of other organisms (such as animals) in the same way.

Now, in eLife, Joaquín Calatayud and co-workers, who are based at Umeå University and Universidad Rey Juan Carlos, report a new set of climate regions based on an analysis of 26,000 species of tetrapods, including birds, amphibians, mammals and reptiles (Calatayud et al., 2021). Tetrapods were chosen for this study because their distributions and evolutionary relationships are well known, and because their home ranges and dispersal abilities are different. To determine the local climate of each species, Calatayud et al. calculated the water level (measured as annual rainfall) and energy level (estimated as the amount of evaporation that occurs each month) of their home range: this means, for example, that a tropical rainforest would be a region with high water and energy levels.

Calatayud et al. hypothesized that the geographical location of different climates accounts for most of the geographical variation in tetrapod distributions. To investigate this, they used a computational analysis tool to cluster together local climates that had similar pools of species, and then mapped each group’s geographical location. This revealed 16 distinct climate regions, some of which differ from the regions classified using the Köppen system.

Three of the mismatches were high-energy regions with warmer temperatures that covered rainforests in the Amazonia, Africa and Southeast Asia, while the other four were lower energy regions that covered areas such as the tundra, montane forests, white mountains, and temperate broadleaf forests. Most of these mismatched regions had different species of tetrapods than the regions in the Köppen system, which suggests that variations in climate may impact the distribution of animals differently to plants. It could also imply that these climate regions impose less restrictions, and allow species with a variety of different adaptations to survive in these conditions.

Common approaches for the classification of climate regions are well suited for detecting abrupt changes in climate (Kottek et al., 2006), but may fail to detect more gradual transitions. Calatayud et al. found that most of the regions they identified had more ‘diffuse’ boundaries and contained tetrapod species from neighboring climate groups. This suggests that variations in climate restricts the dispersal of tetrapods less than plants, with the exception of arid climates, such as deserts and continental polar regions, which showed more abrupt transitions.

Lastly, Calatayud et al. also found that similar climate regions had similar tetrapod populations, even if they were thousands of miles apart (such as the Amazonian and African rainforests), but there were exceptions: for example, species living in the Australian desert differed from those in the Namib desert or Horn of Africa, despite all three locations being deserts with similar climates (Figure 1). This implies that climate alone is insufficient to account for all patterns of species distribution and abundance, and other variables – such as geographical barriers, colonization abilities, or differences in evolutionary history – may also limit the spread of a species.

**Figure 1.**
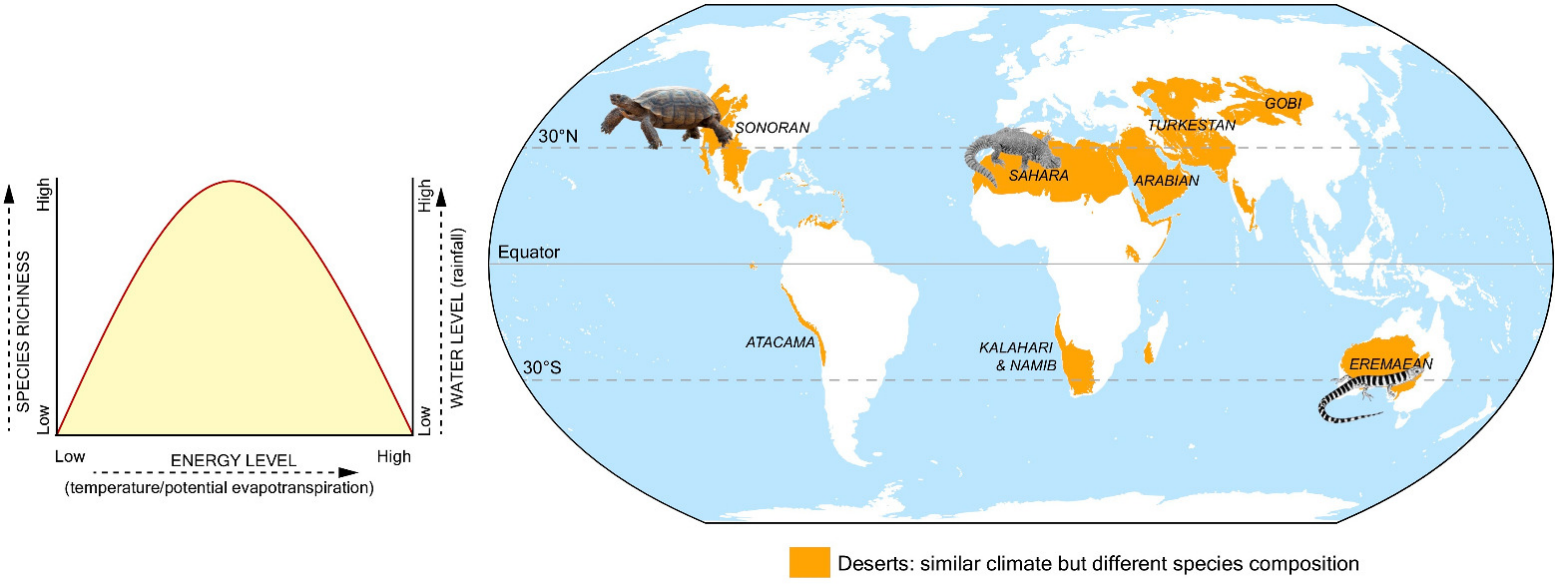
Climate and the distribution of tetrapod species. Calatayud et al. analyzed the climate that 26,000 species of tetrapods experience in their home range, which led to the identification of 16 climate regions. Climates were analyzed in terms of water level (measured as annual rainfall) and energy level (estimated amount of evaporation that occurs each month). Plotting the species richness (y-axis; left), energy (x-axis) and water levels (y-axis; right) in each of these regions reveals that areas with mid-levels of energy and high rainfall have the greatest species richness (left side graph). Calatayud et al. found that similar climate regions tended to have similar tetrapod populations, even if they were thousands of miles apart. However, this was not the case for arid regions such as deserts (shown in orange on the map): for example, bearded lizards that have colonized the Sahara are not found in the Eremaean desert region in Australia, despite both having similar climates.

A number of complications in the analysis will no doubt raise questions. As with all clustering analysis, selecting the optimal number of bins (the number of intervals that the values recorded will be aggregated into) is critical for biogeographic regionalization (Kreft and Jetz, 2013; Daru et al., 2020). An insufficient number of bins can result in the loss of information, whereas an excessive number can compromise model performance (Rosvall and Bergstrom, 2008; Vilhena and Antonelli, 2015). In addition, the analysis only used two climatic variables – water and energy – and other variables may show different results.

Nonetheless, this study challenges the idea of using the same climate classification for multiple organisms, and suggests ecological studies should use systems tailored for each taxonomic group. An exciting avenue of research could be to quantify the phylogenetic and functional differences underlying the climate region of each tetrapod group in comparison to lesser known taxa, such as microbes or insects. Further analyses of climate classification might also help identify climate regions that are the result of human activities, causing organisms to spread into new environments, or native species to go extinct.

These findings have direct implications for our understanding of how tetrapods track climate differently to plants. They may also provide clues as to which animal communities are most susceptible to changes driven by the overall warming of the Earth’s climate.
